# Evidence for validity of five secondary data sources for enumerating retail food outlets in seven American Indian Communities in North Carolina

**DOI:** 10.1186/1479-5868-9-137

**Published:** 2012-11-22

**Authors:** Sheila E Fleischhacker, Daniel A Rodriguez, Kelly R Evenson, Amanda Henley, Ziya Gizlice, Dolly Soto, Gowri Ramachandran

**Affiliations:** 1 Senior Public Health & Science Policy Advisor, NIH Division of Nutrition Research Coordination, National Institutes of Health, US Department of Health and Human Services, Two Democracy Plaza, Room 635, 6707 Democracy Boulevard, MSC 5461, Bethesda, MD, 20892-5461, USA; 2 Department of City and Regional Planning in the College of Arts and Sciences, 319 New East, Chapel Hill, NC, 27514, USA; 3 Department of Epidemiology in the Gillings School of Global Public Health, Center for Health Promotion and Disease Prevention, Bank of America Center, 137 East Franklin St, Suite 306, CB # 8050, Chapel Hill, NC, 27514, USA; 4 Geographic Information Systems Librarian, Reference Department in the Walter Royal Davis Library, 208 Raleigh St CB #3900, Chapel Hill, NC, 27514, USA; 5 Director, Biostatistical Support Unit, Center for Health Promotion and Disease Prevention, 1700 Martin Luther King Jr. Blvd, CB# 7426, Chapel Hill, NC, 27599-7426, USA; 6 Spatial Data Analyst, Center for Health Promotion and Disease Prevention, 319 New East, Chapel Hill, NC, 27514, USA; 7 Healthy Eating Research Assistant, Center for Health Promotion and Disease Prevention, 1700 Martin Luther King Jr. Blvd, CB# 7426, Chapel Hill, NC, 27599-7426, USA

**Keywords:** Food environment, Measurement, Ground-truth, Secondary data, Validity, American Indian, Rurality, Global Positioning Systems (GPS), Geographic Information Systems (GIS)

## Abstract

**Background:**

Most studies on the local food environment have used secondary sources to describe the food environment, such as government food registries or commercial listings (e.g., Reference USA). Most of the studies exploring evidence for validity of secondary retail food data have used on-site verification and have not conducted analysis by data source (e.g., sensitivity of Reference USA) or by food outlet type (e.g., sensitivity of Reference USA for convenience stores). Few studies have explored the food environment in American Indian communities. To advance the science on measuring the food environment, we conducted direct, on-site observations of a wide range of food outlets in multiple American Indian communities, without a list guiding the field observations, and then compared our findings to several types of secondary data.

**Methods:**

Food outlets located within seven State Designated Tribal Statistical Areas in North Carolina (NC) were gathered from online Yellow Pages, Reference USA, Dun & Bradstreet, local health departments, and the NC Department of Agriculture and Consumer Services. All TIGER/Line 2009 roads (>1,500 miles) were driven in six of the more rural tribal areas and, for the largest tribe, all roads in two of its cities were driven. Sensitivity, positive predictive value, concordance, and kappa statistics were calculated to compare secondary data sources to primary data.

**Results:**

699 food outlets were identified during primary data collection. Match rate for primary data and secondary data differed by type of food outlet observed, with the highest match rates found for grocery stores (97%), general merchandise stores (96%), and restaurants (91%). Reference USA exhibited almost perfect sensitivity (0.89). Local health department data had substantial sensitivity (0.66) and was almost perfect when focusing only on restaurants (0.91). Positive predictive value was substantial for Reference USA (0.67) and moderate for local health department data (0.49). Evidence for validity was comparatively lower for Dun & Bradstreet, online Yellow Pages, and the NC Department of Agriculture.

**Conclusions:**

Secondary data sources both over- and under-represented the food environment; they were particularly problematic for identifying convenience stores and specialty markets. More attention is needed to improve the validity of existing data sources, especially for rural local food environments.

## Background

Ecological approaches have helped to elucidate how availability, accessibility, and affordability of healthy and unhealthy foods in the home, school, work, and community are associated with eating patterns [1-3]. The food options available in a neighborhood have been linked to risk for obesity [4], cardiovascular disease [5], and Type 2 diabetes mellitus [6]. Recent initiatives have focused on cataloguing access to food retail outlets, such as the United States Department of Agriculture (USDA) Food Atlas (http://www.ers.usda.gov/foodatlas/) and Food Desert Locator (http://www.ers.usda.gov/data/fooddesert/). Policy initiatives at the local, state, tribal, and federal levels have also targeted improving access to healthy foods in underserved communities [7,8]. Nonetheless, our understanding of how the food environment affects consumer eating behavior and health outcomes is relatively new and findings are mixed [9-11].

The majority of studies examining associations between the local food environment and health behaviors and outcomes have relied on secondary sources, such as the local health department or commercial products, to describe the food environment. Experts in measuring the food environment brought together by the US National Cancer Institute in 2006 recommended future studies evaluate the psychometric properties of secondary retail food data sources, as well as conduct more on-the-ground measures to help develop more valid, reliable, and cost-effective methods of measuring the food environment [12]. Over the last five years, the validity of secondary retail food data sources has been explored in both rural and urban settings, primarily through on-site verification studies [13-20]. While these studies have captured new outlets, most have not involved systematic canvasses of the targeted study area and have tended to focus on outlets and areas identified solely by secondary data sources [14-16,18,21]. Precise Global Positioning Systems (GPS) data were not collected in most of the studies [14-17] and only three used on-site observations of food outlets using GPS without a preconceived notion or list to guide the observations (i.e., “ground-truthing”) [13,22,23]. This ground-truthing approach is considered the gold standard for measuring the food environment since observers are not biased by a list or map of secondary data sources [22,24].

Recent studies have compared multiple sources with GPS data and reported moderate sensitivities, particularly for food establishment information from local health department sources [18,19,23], InfoUSA [19,22], and Dun & Bradstreet [19,22]. Not all of these studies, however, have reported advanced statistical analysis by a single data source (e.g., sensitivity of ReferenceUSA) or by food outlet type (e.g., positive predictive value of Reference USA for convenience stores), hindering our understanding of the validity of a particular data source for accurately identifying a particular food outlet type [13,16,18,25]. Often, these studies provide little detail on secondary data entry and editing, food category classification, or field-based auditing [20,26-28]. Thus, secondary data sources continue to both over- and under-represent the number of food outlets within a study area when compared to field observations.

Further, few food environment assessments have been conducted in American Indian communities, even though American Indians are at increased risk for food insecurity and diet-related chronic diseases [29,30]. More than 550 federally recognized tribes and state recognized tribes are located in the US; not all tribes have a reservation and the US Census estimates that at least 64% of American Indians do not live on reservations [31]. A tribe with federal recognition has petitioned or asked the federal government to recognize or accept their group as a “tribe” and this recognition is only given if certain criteria are met. Three federally recognized tribes in Arizona and New Mexico have been working on healthy store interventions, and they have found that some tribal members travel as far as 30 miles off the reservation to access a diverse supply of affordable, healthy foods [32].

To advance the science on measuring the food environment, we conducted direct, on-site observations of a wide range of food outlets in multiple American Indian communities without a list guiding the field observations, and then compared our findings to several secondary data sources.

## Methods

This work was approved by the Institutional Review Board of the University of North Carolina (NC) at Chapel Hill.

### Study area

The sixth largest population of American Indians in the US and the highest concentration of American Indians east of the Mississippi River reside in NC (http://www.doa.state.nc.us/cia/). The US Census 2010 estimates that 122,110 American Indian/Alaskan Native individuals live in NC. The state is home to eight tribes and four urban Indian organizations. Seven of eight tribes agreed to participate in the American Indian Healthy Eating Project: the Coharie Indian Tribe, Haliwa-Saponi Indian Tribe, Lumbee Tribe of NC, Occanneechi Band of the Saponi Nation, Meherrin Indian Tribe, Sappony, and Waccamaw Siouan Tribe. The one federally recognized tribe in the State, which resides on a reservation, opted out of the study citing existing local efforts to address healthy eating. We did not examine food access for the four urban Indian organizations in NC since there was low American Indian concentration in these four metropolitan areas.

The Census uses State Designated Tribal Statistical Areas (SDTSAs) to represent a compact, contiguous area containing a statistically significant concentration of people who identify with a specific recognized tribe without a reservation and/or residing on off-reservation trust land (http://www.census.gov/geo/www/tsap2010/tsap2010_sdtsa.pdf). We used preliminary 2010 SDTSA maps, available in fall 2009, to determine our study areas. Sappony is physically located in NC and is recognized as a tribe in this state. Sappony is also physically located in Virginia but the state of Virginia has yet to recognize the tribe and Sappony does not have a SDTSA in Virginia. Therefore, for the data validation component of the study, we did not include food data gathered for Sappony in Virginia.

### Secondary data

Using ArcGIS 9.3.1, ZIP Code and county boundaries were overlayed with SDTSA boundaries to identify NC ZIP Codes and counties that intersected or were co-located with the SDTSA. ZIP Codes (n=78) and counties (n=21) co-located with the seven SDTSAs were used to gather information by tribe on food outlets from one free, online directory (online Yellow Pages), two government sources (county health departments and the state agriculture department), and two commercial sources (ReferenceUSA and Dun & Bradstreet).

Our protocol for gathering information from online Yellow Pages was to enter “food” into the search box labeled “find” for each ZIP Code co-locating with each SDTSA. Only outlets physically located within our ZIP Code of interest were included. Food outlets listed in the following categories were included initially and then phone and Internet searches were used to establish all outlets sold food to the public: canners & food processors, convenience stores, fast food restaurants, food and beverage consultants, food banks, food delivery service, food facilities consultants, food processing and manufacturing, food processing equipment and supplies, food products, food products-wholesale, food service management, frozen food locker plants, frozen food, frozen food-wholesale, fruit and vegetable-wholesale, fruit and vegetable markets, grocers-ethnic foods, grocers-specialty foods, grocers-wholesale, grocery stores, health and diet food products, health and diet food products-wholesale, health food restaurants, Mexican food products, natural food, nuts-edible, restaurants, soul food restaurants, and vitamins and food supplements.

For local health county food inspection listings, all co-locating NC counties (n=21) were called in fall 2009. All 21 counties mailed, emailed, or faxed free copies of their latest inspection lists or directed us to a website where their local food inspection data could be accessed and downloaded for free via the Internet. Food outlets listed in the following categories were included initially and phone and Internet searches were used to establish all outlets sold food to the public: food stands, meat markets, mobile food units, pushcarts, and restaurants. For the NC Department of Agriculture and Consumer Services food inspection listings, the Department provided us with an up-to-date listing of all food establishments it inspects within all co-locating NC counties (n=21) in December 2009. Food outlets listed in the following categories were included initially and phone and Internet searches were used to establish all outlets sold food to the public: bakeries, farmers’ markets, and stores with packaged goods sold to the public.

Using our university’s e-research tools, we accessed ReferenceUSA. We conducted a custom search for our selected NAICS codes found within all co-locating NC ZIP Codes (n=78). We gathered all NAICS outlets by ZIP Code. The outlets identified through this search were reviewed and sorted to eliminate or flag any potential questionable food outlets or delete duplicates. Food outlets listed in the following NAICS were included initially and phone and Internet searches were used to establish all outlets sold food to the public: 445 Food and Beverage Sales, 4451 Grocery Stores, 445110 Supermarkets and Other Grocery (except Convenience) Stores, 445120 Convenience Stores, 4452 Specialty Food Stores, 445210 Meat Markets, 445220 Fish and Seafood Markets, 445230 Fruit and Vegetable Markets, 445291 Baked Goods Stores, 445292 Confectionery and Nut Stores, 445299 All Other Specialty Food Stores, 447 Gasoline Stations, 447110 Gasoline Stations with Convenience Stores, 72 Accommodation and Food Services, 722 Food Service and Drinking Places, 7221 Full-Service Restaurants, 722110 Full Service Restaurants, 7222 Limited-Service Eating Places, 722211 Limited-Service Restaurants, 722212 Cafeteria, Grills Buffets, and Buffets, 722213 Snack and Nonalcoholic Beverage Bars, 4299 Other General Merchandise Stores, 452910 Warehouse Clubs and Superstores, 452990 All Other General Merchandise Stores, 452112 Discounted Department Stores, and 446110 Pharmacies and Drug Stores. Using resources from the NC Department of Commerce, Economic Development Intelligence Systems, we accessed without charge Dun & Bradstreet. We conducted a custom search for our selected NAICS codes found within all co-locating NC counties (n=21). We gathered all NAICS outlets by county. Food outlets listed in the same NAICS codes noted above for RefereneUSA were included initially. Phone and Internet searches were used to establish all outlets sold food to the public.

Our general approach was to include any food outlet open and regularly selling publicly accessible food. For each food outlet, we gathered the name, address, city, state, ZIP Code, and phone number. We tracked discrepancies, such as differing names and addresses for outlets determined through phone calls and Internet searches to be the same. Each outlet was viewed in Google Street View, and any differences in name, address, and open/closed status were documented, and then verified through phone calls when possible. We separated conjoined outlets such as KFC/Taco Bell into two outlets. We noted that an outlet was closed if we could verify this in the field, through a phone call with the county health inspector, or a phone call with a new food outlet operating at or near the closed outlet’s location.

Intra-reliability was assessed by comparing the name, address, city, and ZIP Code for all food outlets against each other gathered for four ZIP Codes (n=110; 3% of the final number of secondary food outlets). These four ZIP Codes were co-located with two tribes before they were reconciled into one list per ZIP Code. Then, four reviewers (SF, GR, DS, AR) identified duplicates or non-food sources. Any outlet identified as questionable by the four reviewers was further examined before it was eliminated as a true duplicate, non-food source, or combined and modified to the most accurate name, address, city, state, and ZIP Code available through the phone, online, and community verification processes. Any outlet that was combined with another food outlet, modified, or edited was tracked separately and these changes were tracked by data source and type of changes. For example, if Dun & Bradstreet named a food outlet at 123 Jones Street a McDonald’s while InfoUSA identified a Burger King at a similar address and both data sources were found through phone calls or field observations to be referring to the same fast food outlet currently operating as a McDonald’s at 124 Jones Street, then the two outlets were combined as one food retail listing and the edits made to make this combination of food retail listings were commuted as edits to the secondary data sources. These combinations were not considered “true duplicates”, which we defined as outlets with the same exact name and address. Additional file 1 provides further details on our protocol development for each of the secondary data sources, our secondary data editing steps, and our inter-rater reliability procedures.

In ArcGIS (Esri, Redlands, CA), we used the addresses from secondary data sources and the 2009 TIGER/Line roads data from the Census Bureau to geocode the food sources identified by secondary data (n=3389). The geocoding process assigned geographic coordinates to addresses by matching them with a geospatial database. We were able to geocode 2816 of the 3389 outlets identified (83%). For the remaining unmatched outlets (n=573), we used the Excel Geocoding tool v3.1 from Juice Analytics (http://www.juiceanalytics.com/) and found 336 address-level precision geocodes. We were unable to geocode 237 outlets at the address-level using either geocoding tool. Ultimately, 3152 outlets out of 3389 outlets (93%) were geocoded and included in the analysis.

### Ground-truthing data

To directly observe the food environment, we developed a ground-truthing protocol to drive all roads and streets in each SDTSA (Additional file 2). The Census 2009 TIGER/Line roads data have been shown to be reliable. These road data were used to calculate the road mileage in each SDTSA and create a map of the roads to ground-truth in each SDTSA [33]. The Lumbee Tribe of NC encompasses over 6000 miles, so we worked with the Lumbee Tribal Council and consulted with a demographer to focus on ground-truthing the largest US Census-Designated Place (CDP) in this tribe’s SDTSA with 75% or more American Indian (i.e., Lumberton, NC), along with another CDP with 75% or more of American Indian, considered the “heart” of the tribe where all tribal government and services are located (i.e., Pembroke, NC).

The following types of roads were not driven: private, industrial parks, unpaved, or residential roads such as apartment complexes, residential subdivisions, condominium complexes, and trailer parks. Roads not illustrated on the map but within the SDTSA, while few, were driven and documented by name, and their relative location was noted on the ground-truthing master map. GPS assisted in identifying a few unlabeled or unidentified roads while in the field. Usually, these new roads were small, residential blocks without any food outlets located on them.

We collected the latitude and longitude of each food outlet, completed a short survey of the outlet’s location and food classification, and used photography to help capture the outlet’s location and food classification. Outlets that appeared closed or had signs indicating that they were under renovation or coming soon were also captured. We determined whether these stores were in business through Internet searches, phone calls, re-visiting the area, or during the inter-rater reliability testing. Primary data collection was conducted from February through June 2010. Two independent research assistants (JSR, DS) conducted an inter-rater reliability process of our ground-truth protocol in September-October 2010 by driving 10% of all roads within the SDTSA for six of the tribes and 10% of all roads within Lumberton. GPS data were uploaded into Google Earth and then converted to a shapefile in ArcGIS using the Arc2Earth extension. A distance of 1600 meters was used to compare the outlets identified during the inter-rater process to the outlets identified during the primary ground-truthing data collection. Matches were determined by name. Minor reconciliations were made to differences in names between primary ground-truthed and inter-rater reliability data.

### Categorizing the food outlets

Food outlet types identified by both secondary and ground-truthing were consolidated into six categories: (1) convenience stores, (2) general merchandise stores (e.g., dollar stores and discount department stores, such as Kmart, Target, and Wal-Mart, without a full grocery section), (3) grocery stores, (4) specialty markets & shops (e.g., meat markets, produce stands, bakeries, donut shops, and ice cream shops), (5) restaurants (e.g., fast food, full-service, and coffee shops), and (6) food banks and community gardens. To assist in classifying the secondary data, Internet searches were conducted, phone calls were made to questionable outlets, and experiential knowledge was utilized. During ground-truthing, information to classify chain food outlets was generally gathered from outside of the food outlet; for non-chain food outlets researchers generally went into the outlet and asked a store employee information about the foods sold and, for restaurants, the type of service provided. For some convenience stores in rural areas, researchers asked if gas was currently sold at the location.

To classify food outlets identified through secondary data sources or ground-truthing, we modified the Nutrition Environment Measurement Survey (NEMS) food store and restaurant classification codes [34,35]. We used “other” to capture outlets not easily described with our modified NEMS codes. For restaurants, we used one or more of the following to describe the type of service provided: fast food restaurant (e.g., limited service, counter-only, McDonald’s); fast-casual restaurant (e.g., order at counter but delivered to your table, Corner Bakery); full-service restaurant (e.g., waiter comes to your table and takes your order); buffet-style restaurant (e.g., all you can eat buffet option); banquet (e.g., weddings, special events); catering (e.g., bring food to you); delivery (e.g., pizza); and to-go or drive-thru (e.g., pick up and go). Additional file 2 provides the complete list of food codes used in our study and also explains other approaches we used to classify the food outlets [13,34,35]. Inter-rater reliability for classifying all food outlets identified through secondary data sources and through ground-truthing was assessed by comparing percent agreement between two-raters for our modified NEMS and six category food classification coding system used for statistical analyses for all identified outlets.

### Categorizing the level of urbanization

Using 2000 Rural–Urban Commuting Area (RUCA) codes obtained from the US Department of Agriculture, each outlet identified was categorized by its ZIP Code [36]. Similar to other consolidations [19,37], the 10-tiered RUCA system was consolidated into four levels: urban (RUCA 1), sub-urban (RUCA 2), large town (RUCA 3), and small town/rural (RUCA 4).

### Matching ground-truthed data to secondary data

The ground-truthed and secondary data were merged into a single file. The point distance tool in ArcGIS was used to calculate the distance between all outlets identified in secondary data within 1600 meters of outlets identified in ground-truthed data. Internet searches and phone calls were made to confirm matches for convenience stores, diners, and smaller, non-chain venues that were questionably similar but not exact matches in name or relative distance. We also explored possible matches with secondary data that did not geocode or were not within 1600 meters of the ground-truthed outlet. In ArcGIS, we used the select-by-location tool to identify outlets that fell within the boundaries of the six SDTSAs and the two CDPs examined, excluding secondary data outlets identified outside of the SDTSA.

### Analysis

Sensitivity, kappa, positive predictive value (PPV), and concordance were calculated to assess the validity of secondary data sources. These were interpreted using the Landis and Koch criteria (<0.00 poor, 0.00-0.20 slight, 0.21-0.40 fair, 0.41-0.60 moderate, 0.61-0.80 substantial, and 0.81-1.00 almost perfect) [38]. Sensitivity was calculated as the ratio of the number of ground-truthed outlets that matched secondary data outlets to the number of ground-truthed outlets that matched secondary data outlets plus the number of ground-truthed food outlets that did not match secondary data outlets. PPV was calculated as the proportion of the establishments listed by the secondary data sources that were observed on the ground. Concordance was calculated as the proportion of the establishments observed on the ground and listed by the secondary data sources among all the establishments either on the ground or listed. We calculated 95% confidence intervals for each of these proportions by approximating the binomial distribution with a normal distribution. Analyses were conducted using SAS software (version 9.2; SAS Institute, Inc., Cary, NC).

## Results

### Intra- and inter-rater reliability

Intra-rater reliability for data entry was 100%, determined by comparing the name, address, city, and ZIP Code for all food outlets gathered for four ZIP Codes (n=110; 3% of the final number of secondary food outlets).

Approximately 144 miles were driven during the inter-rater reliability phase and 219 food outlets were identified. One outlet was deleted since it was not open at the times when the inter-rater reliability team or the primary data collection team was in the field. An additional three outlets were excluded since they were not considered food outlets. A total of 203 outlets matched the primary ground-truth data (94%). The average distance for 202 outlets between the latitudes and longitudes taken during primary data collection and compared to the inter-rater reliability team was 32 meters, with a range from 0 to 1418 meters (standard deviation 142). When excluding three outliers (from a large farm, winery, and strip mall gas station) the standard deviation was 16 meters. As a result of the inter-rater reliability process, 12 new outlets were added to the comprehensive food list; since they were outlets in the areas examined but were not captured during the primary data collection process. There were several possible reasons for the additions: seasonal produce stands (n=2), outlets may have not been open when the primary data were collected (n=2), the primary data collection team might not have been able to determine if the outlet sold food (n=2), and one outlet was a mobile food vendor that likely was not in the area during the primary data collection (n=1). The inter-rater reliability process did not identify eight outlets found during the primary data collection. All of these outlets were in three urban settings and tended to be small convenience stores or grill venues. Therefore, after including the additional 12 new outlets to the analysis, the overall percent agreement between the inter-rater reliability team and the primary data team for the areas canvassed was 91%.

Percent agreement between two raters for our six-category food classification coding system was 100%. The inter-rater reliability process for food classifications using the modified NEMS classification in ground-truthing was 94% (202 outlets were classified the same, out of 215 outlets compared). Minor differences were generally between mixed American restaurant/diners, grills, and bakeries. There was 100% agreement on food service style for all restaurants.

### Outlets edited and identified

Almost a quarter of the secondary data retail food outlets (24% or 827 outlets of the 3434 secondary outlets gathered) examined were determined to be the same outlets, despite slightly different contact information from the five secondary data sources. That is, 1244 differences in name, address, city, and ZIP Code were identified among and between the data sources in referring to the same food outlet. These were not the “true” duplicates, which we eliminated based on the same name, address, city, state, and ZIP Code. The information on 162 of these outlets was changed based on the Google Street View review process. We were not able to view 199 of the outlets identified through secondary data or ground-truthing in Google Street View (22%).

We drove over 1,502 miles and identified 699 food outlets while ground-truthing (Table 1). The road data guided us through our six SDTSA and two CDPs; only a small percentage of street names were missing on short rural or residential roads. The few rural, often unpaved roads we did not drive and excluded while in the field did not have any secondary food outlets located near them. Based on community input, our on-site viewing, and virtual viewing through multiple online imagery views, these few un-driven roads did not have any sign of commercial activity. Based on the food outlet names recorded while ground-truthing, the names of 42 food outlets identified by secondary data sources were changed (7% of the 564 ground-truthed/secondary food outlet matches). The majority of these name changes were for convenience stores (55%). The food classifications of 80 outlets in the secondary data were modified based on information gathered through ground-truthing; which provided more detail on the type of restaurant or convenience store. The average distance between the latitudes and longitudes taken for 531 ground-truthed food outlets and secondary data food outlets was 198 meters, with a range from 3 to 1496 meters and a standard deviation of 267. Thirty-three outlets were matched only by name and city, because secondary data fell outside of the 1600 meter match buffer, or the outlets could not be geocoded.

**Table 1:** Geographical and ground-truthing descriptions for each of the seven participating American Indian tribes, 2009-2010

	**Sappony**	**Waccamaw Siouan Tribe**	**Occaneechi Band of the Saponi Nation**	**Meherrin Indian Tribe**	**Haliwa-Saponi Indian Tribe**	**Coharie Indian Tribe**	**Lumbee Tribe of North Carolina**	**Totals**
ZIP Codes overlaying with SDTSA^1^	1	7	5	4	10	8	43	**78**
Counties overlaying with SDTSA^1^	1	2	3	2	5	3	9	**21**^**2**^
Total miles of roadways within SDTSA^3^	100	97	135	110	305	455	238^4^	**1440**
Miles covered ground-truthing within SDTSA	116	43	120	90	275	659	199^4^	**1502**
Days spent conducting primary ground-truthing data collection	1	1	2	2	2	7	5	**20**
Outlets identified through primary ground-truthing	5	6	13	62	31	315	234	**666**
Additional outlets identified through ground-truthing for inter-rater reliability analysis^5^	0	0	0	2	1	5	4	**12**
Closed outlets	0	0	1	2	3	7	8	**21**
Total outlets	5	6	14	66	35	327	246	**699**

^1^Using ArcGIS 9.3.1, ZIP Code and county boundaries were overlayed with State Designated Tribal Statistical Area (SDTSA) boundaries to determine NC ZIP Codes and counties intersected or co-located with the SDTSA.

^2^Four counties co-located with two tribes and were therefore only counted once in the total county count.

^3^Determined in ArcGIS using TIGER/Line 2009 summaries of all primary (S1100), secondary (S1200), and local (S1400) roads.

^4^Only focused on two United States Census Designated Places with the SDTSA.

^5^10% of six of the SDTSA areas and one of the two United States Census Designated Places was driven during the inter-rater reliability process; one area was driven over two visits while the rest was driven during the one and only visit.

### Matches and analysis

The majority of outlets observed in the field while ground-truthing matched data from at least one of the five secondary data sources (n=564, 83%) (Table 2). Some matches, however, differed by type of food outlet observed; the highest match rates were found in grocery stores (97%), general merchandise stores (96%), and restaurants (91%), while lower match rates were observed in convenience stores (76%) and specialty markets and shops (52%). Similar match rates were observed in our four levels of urbanization, ranging from 80-89%. A total of 114 ground-truthed outlets did not match any of the five secondary data outlets; they were primarily convenience stores (24%) and specialty markets and shops (48%). Only a few restaurants (n=29, 9%) did not match at least one secondary data source. 

**Table 2:** Percent and number of matches* for opened^ food outlets between ground-truthed data and five secondary food retail data sources in six State Designated Tribal Statistical Areas (SDTSA) and two United States Census-Designated Places in North Carolina, 2009-2010 (n=870)

	**Ground-truthed (n=678)**	**All Secondary Retail Food Data Sources**^**1 **^**(n=756)**	**Local Health County (n=438)**	**State Agriculture Department (n=125)**	**ReferenceUSA (n=597)**	**Dun & Bradstreet (n=272)**	**Online Yellow Pages (n=398)**
	**Percent of matches (number of matches/total number of food outlets)**						
**Overall**	83 (564/678)	75 (564/756)	85 (372/438)	78 (97/125)	84 (505/597)	86 (235/272)	77 (308/398)
**By Type of Food Outlet**							
Convenience Stores	76 (164/215)	74 (164/222)	81 (58/72)	79 (42/53)	80 (141/176)	78 (53/68)	73 (79/108)
General Merchandise^2^	96 (43/45)	86 (43/50)	0 (0/1)	80 (8/10)	87 (41/47)	95 (37/39)	82 (9/11)
Grocery Stores	97 (32/33)	89 (32/36)	97 (29/30)	100 (28/28)	94 (32/34)	93 (26/28)	100 (22/22)
Specialty Markets & Shops^3^	52 (31/60)	48 (31/64)	83 (15/18)	50 (11/22)	64 (21/33)	60 (6/10)	53 (18/34)
Restaurants^4^	91 (294/323)	77 (294/383)	85 (270/317)	67 (8/12)	88 (270/306)	89 (113/127)	81 (180/223)
Food Bank	0 (0/2)	0 (0/1)	0 (0/0)	0 (0/0)	0 (0/1)	0 (0/0)	0 (0/0)
**By Level of Urbanization**							
Urban	89 (8/9)	73 (8/11)	75 (3/4)	67 (2/3)	100 (6/6)	100 (2/2)	50 (1/2)
Sub-Urban	80 (70/87)	81 (70/86)	92 (46/50)	90 (9/10)	91 (64/70)	100 (22/22)	80 (36/45)
Large Town	85 (268/314)	76 (268/354)	84 (179/213)	84 (47/56)	84 (241/287)	83 (114/138)	76 (122/160)
Small Town & Rural	81 (218/268)	71 (218/305)	84 (144/171)	70 (39/56)	83 (194/234)	88 (97/110)	78 (149/191)

*Matches were determined as follows: For ground-truthed and all secondary retail food data sources, a match occurred if a ground-truthed outlet matched any secondary food retail data source. For each of the five individual secondary food retail data sources, a match occurred if a ground-truthed outlet matched that particular secondary data source.

^Outlets determined to be closed were excluded from the matching analyses.

^1^All secondary retail food data sources combined, ranging from one source identifying outlet to all five sources, within the SDTSA.

^2^Includes dollar stores and discount department stores that do not have a full grocery section, such as Kmart, Target, and Wal-Mart.

^3^Includes meat markets, produce stands, bakeries, donuts, and ice cream.

^4^Includes fast food, full-service, and coffee shops.

Overall, ReferenceUSA exhibited almost perfect sensitivity (0.89) (Table 3). Local health department data had substantial sensitivity (0.66) and was almost perfect when focusing only on restaurants (0.91), for which they monitor by law. The remaining three data sources had lower sensitivity: online Yellow Pages (0.55), Dun & Bradstreet (0.41), and the state agriculture department (0.17). Overall, PPV was substantial for ReferenceUSA (0.67) and moderate for local health departments (0.49). Overall, concordance was moderate for both ReferenceUSA (0.57) and local health departments (0.42). Overall, kappa statistics were substantial for ReferenceUSA (0.62), moderate for local health departments (0.41), fair for Dun & BradStreet (0.24) and online Yellow Pages (0.24), and slight for the state agriculture department (0.06).

**Table 3:** Evidence for validity of five data sources for enumerating retail food outlets in comparison to ground-truthed data for open and closed outlets in six State Designated Tribal Statistical Areas and two US Census-Designated Places in North Carolina, 2009-2010

**Secondary Data Source**	**Type of Food Outlet (n=total number of food outlets by outlet type)**			
	**All Food Outlets (n=891)**			
	**Agreement Statistics (95% Confidence Interval)**			
	Sensitivity (95% CI*)	Positive Predictive Value (95% CI*)	Concordance (95% CI*)	Kappa (95% CI*)
Local Health County	0.66 (0.62, 0.70)	0.49 (0.46, 0.53)	0.42 (0.39, 0.46)	0.41 (0.36, 0.47)
State Agriculture Department	0.17 (0.14, 0.20)	0.13 (0.10, 0.15)	0.11 (0.09, 0.13)	0.06 (0.03, 0.10)
ReferenceUSA	0.89 (0.86, 0.92)	0.67 (0.63, 0.70)	0.57 (0.54, 0.61)	0.62 (0.56, 0.67)
Dun & Bradstreet	0.41 (0.37, 0.45)	0.31 (0.28, 0.34)	0.27 (0.24, 0.30)	0.24 (0.20, 0.29)
Online Yellow Pages	0.55 (0.51, 0.59)	0.41 (0.38, 0.45)	0.35 (0.32, 0.39)	0.24 (0.18, 0.30)

*(lower 95% confidence interval, upper 95% confidence interval).

ReferenceUSA had the highest sensitivity for convenience stores (0.86), but relatively low sensitivity for other food outlet types (Table 4). For general merchandise stores, local health departments (0.97), ReferenceUSA (0.95), and Dun & Bradstreet (0.86) had almost perfect sensitivity. All five sources had substantial to almost perfect sensitivity for grocery stores, ranging from 0.68 (online Yellow Pages) to 0.94 (ReferenceUSA). Specialty markets and shops had the lowest sensitivity scores for each of the sources, ranging from 0.19 (Dun & BradStreet) to 0.66 (ReferenceUSA). For restaurants, local health departments and ReferenceUSA had similar sensitivity (0.91). PPV was substantial to almost perfect for general merchandise stores for local health departments (0.86), ReferenceUSA (0.82), and Dun & Bradstreet (0.74). For all five data sources, PPV ranged from 0.61 (online Yellow Pages) to 0.89 (ReferenceUSA). For restaurants, PPV was substantial for ReferenceUSA (0.70) and local health departments (0.70). Concordance was lower for convenience stores, ranging from 0.19 (Dun & Bradstreet) to 0.51 (ReferenceUSA), as well as for specialty markets and shops, ranging from 0.06 (Dun & Bradstreet) to 0.22 (ReferenceUSA). Kappa statistics were highest for ReferenceUSA for convenience stores (0.56) and restaurants (0.64). Local health departments were moderate for both grocery stores (0.60) and restaurants (0.56).

**Table 4:** By type of food outlet, evidence for validity of five data sources for enumerating retail food outlets in comparison to ground-truthed data for open and closed outlets in six State Designated Tribal Statistical Areas and two US Census-Designated Places in North Carolina, 2009-2010 (PPV stands for Positive Predictive Value)

**Secondary Data Source**	**Type of Food Outlet (n=total number of food outlets by outlet type)**																			
	**Convenience Stores (n=277)**				**General Merchandise Stores**^**1**^**(n=52)**				**Grocery Stores (n=37)**				**Specialty Markets & Shops2 (n=94)**				**Restaurants3 (n=428)**			
	Sensitivity (95% CI*)	PPV (95% CI*)	Concordance (95% CI*)	Kappa (95% CI*)	Sensitivity (95% CI*)	PPV (95% CI*)	Concordance (95% CI*)	Kappa (95% CI*)	Sensitivity (95% CI*)	PPV (95% CI*)	Concordance (95% CI*)	Kappa (95% CI*)	Sensitivity (95% CI*)	PPV (95% CI*)	Concordance (95% CI*)	Kappa (95% CI*)	Sensitivity (95% CI*)	PPV (95% CI*)	Concordance (95% CI*)	Kappa (95% CI*)
Local Health County	0.35	0.26	0.21	0.20	0.98	0.86	0.83	-0.04	0.91	0.81	0.78	0.60	0.47	0.23	0.16	0.47	0.91	0.70	0.64	0.56
	(0.28, 0.43)	(0.20, 0.32)	(0.16, 0.26)	(0.12, 0.29)	(0.88, 0.99)	(0.73, 0.94)	(0.70, 0.92)	(-0.10, 0.03)	(0.75, 0.98)	(0.64, 0.92)	(0.62, 0.90)	(0.24, 0.96)	(0.29, 0.65)	(0.14, 0.35)	(0.09, 0.25)	(0.28, 0.66)	(0.87, 0.94)	(0.66, 0.75)	(0.60, 0.69)	(0.48, 0.65)
State Agriculture Department	0.26	0.19	0.15	0.14	0.19	0.16	0.15	-0.01	0.88	0.78	0.76	-0.14	0.34	0.17	0.12	0.18	0.03	0.02	0.02	0.00
	(0.19, 0.33)	(0.14, 0.25)	(0.11, 0.20)	(0.06, 0.22)	(0.08, 0.33)	(0.07, 0.29)	(0.07, 0.28)	(-0.14, 0.11)	(0.71, 0.97)	(0.61, 0.90)	(0.59, 0.88)	(-0.23, -0.04)	(0.19, 0.53)	(0.09, 0.28)	(0.06, 0.20)	(-0.03, 0.39)	(0.12, 0.05)	(0.01, 0.04)	(0.01, 0.04)	(-0.02, 0.02)
Reference USA	0.86	0.64	0.51	0.56	0.95	0.82	0.79	0.35	0.94	0.89	0.87	-0.07	0.66	0.32	0.22	0.46	0.91	0.71	0.64	0.64
	(0.80, 0.91)	(0.57, 0.70)	(0.45, 0.57)	(0.46, 0.66)	(0.84, 0.99)	(0.69, 0.91)	(0.65, 0.89)	(-0.01, 0.70)	(0.08, 0.99)	(0.74, 0.97)	(0.71, 0.96)	(-0.14, -0.00)	(0.47, 0.81)	(0.21, 0.45)	(0.14, 0.32)	(0.27, 0.65)	(0.88,0.94)	(0.66, 0.75)	(0.60, 0.69)	(0.56, 0.72)
Dun & Bradstreet	0.32	0.24	0.20	0.17	0.86	0.74	0.71	0.54	0.81	0.72	0.70	0.31	0.19	0.09	0.06	0.15	0.38	0.29	0.27	0.19
	(0.25, 0.40)	(0.18, 0.30)	(0.15, 0.24)	(0.08, 0.26)	(0.72, 0.95)	(0.60, 0.85)	(0.57, 0.83)	(0.26, 0.82)	(0.64, 0.93)	(0.55, 0.86)	(0.53, 0.84)	(-0.06, 0.68)	(0.07, 0.36)	(0.04, 0.19)	(0.02, 0.13)	(-0.03, 0.32)	(0.32, 0.44)	(0.25, 0.34)	(0.22, 0.31)	(0.13, 0.25)
Online Yellow Pages	0.48	0.36	0.29	0.21	0.21	0.18	0.17	0.00	0.69	0.61	0.60	-0.22	0.59	0.29	0.20	0.33	0.61	0.47	0.43	0.23
	(0.40, 0.56)	(0.29, 0.42)	(0.23, 0.34)	(0.10, 0.31)	(0.10, 0.36)	(0.09, 0.31)	(0.08, 0.30)	(-0.13, 0.12)	(0.50, 0.84)	(0.44, 0.77)	(0.42, 0.75)	(-0.37, 0.07)	(0.41, 0.76)	(0.19, 0.42)	(0.13, 0.30)	(0.13, 0.53)	(0.56, 0.67)	(0.42, 0.52)	(0.38, 0.48)	(0.14, 0.32)

^1^Includes dollar stores and discount department stores that do not have a full grocery section, such as Kmart, Target, and Wal-Mart.

^2^Includes meat markets, produce stands, bakeries, donuts, and ice cream.

^3^Includes fast food, full-service, and coffee shops.

*(lower 95% confidence interval, upper 95% confidence interval).

ReferenceUSA had almost perfect sensitivity in sub-urban, large town, and small town/rural areas (0.87 to 0.92) (Table 5). Online Yellow Pages had moderate to substantial sensitivity, but showed the most variability by levels of urbanization, with a range from 0.46 to 0.69. Sensitivity was generally substantial for local health departments (0.65 to 0.66), fair to moderate for Dun & Bradstreet (0.31 to 0.44), and slight for the state agriculture department (0.12 to 0.18). PPV was substantial for ReferenceUSA in sub-urban, large town, and small town/rural areas (0.63 to 0.75). Dun & Bradstreet had fair PPV in sub-urban, large town, and small town/rural areas (0.25 to 0.32). Concordance was moderate for ReferenceUSA in large towns (0.60) and small town/rural areas (0.54), and substantial in sub-urban areas (0.62). The four other data sources had comparatively lower concordance. Kappa statistics for ReferenceUSA were moderate (0.59) in large town and substantial in small town/rural (0.61) and sub-urban areas (0.74). The other four sources generally exhibited slight to moderate agreements in small town/rural, large town, and sub-urban areas. Our evidence for validity analyses were limited in the urban category (n=12).

**Table 5:** By level of urbanization, evidence for validity of five secondary retail food data sources for enumerating retail food outlets in comparison to ground-truthed data for open and closed outlets in six State Designated Tribal Statistical Areas and two US Census-Designated Places in North Carolina, 2009-2010 (PPV stands for Positive Predictive Value)

**Secondary Data Source**	**Level of Urbanization (n=total number of food outlets by outlet type)**															
	**Small Town & Rural (n=364)**				**Large Town (n=410)**				**Sub-Urban (n=105)**				**Urban (n=12)**			
	**Agreement Statistics (95% Confidence Interval)**															
	Sensitivity (95% CI*)	PPV (95% CI*)	Concordance (95% CI*)	Kappa (95% CI*)	Sensitivity (95% CI*)	PPV (95% CI*)	Concordance (95% CI*)	Kappa (95% CI*)	Sensitivity (95% CI*)	PPV (95% CI*)	Concordance (95% CI*)	Kappa (95% CI*)	Sensitivity (95% CI*)	PPV (95% CI*)	Concordance (95% CI*)	Kappa (95% CI*)
Local Health County	0.65	0.47	0.40	0.44	0.67	0.51	0.44	0.38	0.66	0.54	0.45	0.47	0.38	0.27	0.25	0.10
	(0.59, 0.72)	(0.41, 0.53)	(0.35, 0.45)	(0.35, 0.52)	(0.61, 0.72)	(0.46, 0.56)	(0.40, 0.49)	(0.30, 0.47)	(0.54, 0.77)	(0.43, 0.65)	(0.35, 0.55)	(0.32, 0.63)	(0.08, 0.76)	(0.06, 0.61)	(0.06, 0.57)	(-0.39, 0.59)
State Agriculture Department	0.18	0.13	0.11	0.05	0.17	0.13	0.12	0.08	0.13	0.10	0.09	0.07	0.25	0.18	0.17	0.00
	(0.13, 0.23)	(0.09, 0.17)	0.08, 0.14	(-0.02, 0.11)	(0.13, 0.22)	(0.10, 0.17)	(0.09, 0.15)	(0.03, 0.12)	(0.06, 0.23)	(0.05, 0.19)	(0.04, 0.16)	(-0.00, 0.14)	(0.03, 0.65)	(0.02, 0.52)	(0.02, 0.48)	(-0.44, 0.44)
ReferenceUSA	0.88	0.63	0.54	0.61	0.90	0.68	0.60	0.59	0.92	0.75	0.62	0.74	0.75	0.55	0.50	-0.29
	(0.83, 0.92)	(0.58, 0.68)	(0.48, 0.59)	(0.52, 0.69)	(0.86, 0.93)	(0.63, 0.73)	(0.55, 0.64)	(0.50, 0.67)	(0.83, 0.97)	(0.64, 0.83)	(0.52, 0.71)	(0.60, 0.88)	(0.35, 0.97)	(0.23, 0.83)	(0.21, 0.79)	(-0.62, 0.04)
Dun & Bradstreet	0.44	0.32	0.27	0.31	0.42	0.32	0.28	0.20	0.31	0.25	0.21	-0.62	0.25	0.18	0.17	-0.67
	(0.38, 0.51)	(0.27, 0.37)	(0.22, 0.32)	(0.23, 0.38)	(0.36, 0.48)	(0.27, 0.37)	(0.24, 0.32)	(0.12, 0.27)	(0.20, 0.43)	(0.17, 0.36)	(0.14, 0.30)	(-0.75, -0.49)	(0.03, 0.65)	(0.02, 0.52)	(0.02, 0.48)	(-1.11, -0.22)
Online Yellow Pages	0.69	0.49	0.42	0.38	0.46	0.35	0.30	0.15	0.52	0.42	0.35	0.22	0.12	0.09	0.08	-0.91
	(0.62, 0.74)	(0.44, 0.55)	(0.37, 0.47)	(0.28, 0.47)	(0.40, 0.52)	(0.30, 0.40)	(0.26, 0.35)	(0.07, 0.23)	(0.40, 0.64)	(0.32, 0.54)	(0.26, 0.45)	(0.05, 0.38)	(0.00, 0.53)	(0.00, 0.41)	(0.00, 0.38)	(-0.49, 0.31)

*(lower 95% confidence interval, upper 95% confidence interval).

## Discussion

Despite the importance of characterizing local food environments, only sparse and conflicting information is available about the evidence for validity of secondary food data sources in identifying food outlets. Understanding the local food environment in underserved communities is particularly important since these communities are disproportionately affected by diet-related chronic diseases. In our study, we found relatively high sensitivity for ReferenceUSA and local health department data gathered from 21 counties, particularly for restaurants. Interestingly, even though Dun & Bradstreet is used in many large cross-sectional studies and by the US Centers for Disease Control and Prevention and the USDA Food Atlas and Food Desert Locator, we found it to have relatively low sensitivity (0.41) [39-41].

Our findings were similar to the one other study that used ground-truthed observations to evaluate the evidence for validity of InfoUSA and Dun & Bradstreet data [22]. Our levels of agreement may be higher in part because we did extensive editing to eliminate duplicates and potential problematic outlets before analyzing the data. Our sensitivity and PPV findings are also similar to those of one of the most recent and comprehensive on-site verification studies of secondary food data [19]. In one urban and seven rural counties in South Carolina, Liese et al. [19] conducted an on-site verification with GPS to verify the presence and location of every food outlet on a comprehensive list based on data from local health inspection registers, Dun & Bradstreet, and InfoUSA. On-site verification identified significantly more outlets then any of the three secondary sources. Calculating sensitivity as the fraction of open food outlets listed that were found to be open (“located and open”)/(“located and opened” + “food, not listed”), sensitivities were moderate for local health department data (68%), InfoUSA (65%), and Dun & BradStreet (55%). Two other on-site verification studies with GPS reported higher sensitivity for government food inspection data—specifically, 84% for Newcastle City Council in the United Kingdom [17] and 87% for Albany, New York-inspected food stores [18]. In an earlier study, Paquet et al.[16] did not use GPS in an on-site verification study of 12 census tracts in the Montreal metropolitan region but also reported higher sensitivity on food outlets for the commercial database Tamec Inc. (range: 0.67-0.85) than for an Internet-based list (range: 0.55-0.79) [16]. Sensitivity in these verification studies was probably over-estimated, however, because the denominator was probably smaller than if a ground-truthed approach had been used to systematically canvass all roads in the study area, not just verify outlets identified by secondary sources. Taking this limitation into account, there is increasing evidence in both rural and urban settings of the relatively high sensitivity of ReferenceUSA and local health department data.

Combining ReferenceUSA and local health department data, at least in the NC counties examined, may be useful if physical observations are not feasible. Governments like the State of NC may consolidate local health data into a state database. However, we obtained information from local county health departments rather than the state agency since this approach enabled us to build relationships with inspectors with the most reliable knowledge of the food lists and food environment [42].

Given the time and expense associated with obtaining and editing data from multiple sources, investigators should consider the best source for data to address their study questions. The cost-effectiveness of multiple secondary data sources or a combination of ground-truthing and secondary data needs to be evaluated. Particularly for larger areas, future studies could ground-truth sub-sets of their study area to elucidate the validity of secondary data for their study areas.

To our knowledge, this study along with two others [13,22] are the only validation studies in the US that conducted an independent assessment of the study area using reliable road data and then compared findings to secondary data sources [13]. This approach is considered the gold standard for identifying food outlets [24]. Our study also used precise GPS locational point data, like more recent validation investigations [18,19]. Our entire data collection, including inter-rater reliability assessment, took less than a year, which is comparable to the 10-month range reported in Liese et al. [19]. In our case, we did not ground-truth an area until we had tribal permission to begin. Based on our inter-rater reliability findings, a single observation should be sufficient to characterize a study area, but further work is needed to clarify the temporal stability of food quality and price [43].

Identifying matches between the ground-truthed data and secondary data was difficult, particularly for convenience stores and small diners, given name differences between the data sources and the limited utility of Google Street View and Google Earth in rural settings [44-46]. Unlike Liese et al.[19], we did not assess the geospatial accuracy of our secondary sources, since only two of the five had geospatial data (ReferenceUSA and Dun & Bradstreet) and 7% of our outlets failed to geocode. Like Liese et al. [19], we significantly edited the secondary data prior to comparing the final secondary data list with ground-truth data (24% of the outlets analyzed were combinations of slightly different food outlet information). These editing efforts may have reduced our over-count error. However, while we did not track the time vested in data editing, future investigations should consider not only the cost of secondary data but also the staff time in data editing and auditing when weighing the cost of secondary data against ground-truthing.

We found that 20 days spent ground-truthing our seven study areas to be a worthwhile investment given the under- and over-counting of each of the five secondary data sources. Thus, measuring the food environment should significantly improve with GPS-assisted on-site observations; however, it is not clear whether verifying a comprehensive list of secondary data or ground-truthing a specified area without a preconceived notion of the food environment is the better approach to examining the food environment. We also have only limited understanding of the potential of the USDA’s online tools, Food Environment Atlas and Food Desert Locator, as well as other emerging databases and modeling tools.

In our study, agreement statistics varied by type of food outlet. Powell et al.[22] and Liese et al.[19] also reported that evidence for validity varied across a range of food outlet categories and even within the restaurant category (i.e., full-service versus fast food). Over- and under-count errors associated with type of food outlet have been reported previously [13,19]. In our study, convenience stores and specialty markets tended to have lower evidence for validity for enumerating food retail outlets than restaurants and grocery stores. Convenience store varied widely, from gas stations with convenience stores to chain and local pharmacies with food, to country stores. Likewise, specialty markets and shops included produce stores, meat markets, and ice cream shops. The majority of the restaurant and grocery store categories were chain outlets, generally tracked consistently by local health departments and ReferenceUSA.

Few studies have evaluated the validity of secondary data sources at varying levels of urbanization. To our knowledge, two studies provide the most comprehensive analyses across levels of urbanization [19,22]. The South Carolina study reported no marked differences but only included one urban county to contrast with seven rural counties [19]. The metropolitan Chicago study found few significant differences across levels of urbanization, but convenience store and fast food restaurant agreement was lower in rural areas [22]. Our study sample did not provide an adequate urban sample size for comparisons. Across three levels of urbanization, however, we did not find notable differences between data sources or by food type, except for online Yellow Pages, whose validity ranged from 0.46 to 0.69.

This is one of only two studies to rigorously examine the food environment in American Indian communities. A recent study examined the number and type of food stores—limited to convenience, grocery, and supermarket stores—on federal reservations in Washington State [47]. Stores were identified by telephone survey of tribal headquarters, ReferenceUSA, and on-site visitation using GPS. All stores found in ReferenceUSA were located except for two, and an additional 15 stores were identified during the on-site verification. For the 22 tribes explored, a total of 50 stores were identified. No supermarkets were identified within 17 of the reservations examined. Prior work has also noted the problems that three federally recognized tribes in Arizona and New Mexico have in accessing healthy, affordable foods [32]. The tribes examined in this study are not federally recognized and do not live on reservations. To our knowledge, our study is also the only description of local food environments using spatial data for American Indians living off reservations, though they are estimated to make up more than 60% of the population. The SDTSA used here encompassed varying levels of urbanization, sometimes including local town shopping areas and sometimes not. SDTSA are not discrete counties, Census tracts, or Census block groups, but they provide insights into local rural areas, where non-American Indians live as well. Our findings may thereby have limited generalizability for American Indians living on reservations. Since American Indians are at elevated risk of childhood obesity [48] and Type 2 diabetes [49], more examinations of the food environment within American Indian communities is needed—on reservations, particularly those more geographically isolated, and in off-reservation settings where the majority of American Indians currently live. Further work is also needed to understand the unique land use authority that federally and state recognized tribes have to affect the establishment and regulation of food outlets within their jurisdictions.

Continued efforts to measure the food environment could facilitate sharing of common metrics for deciding study areas, editing secondary data sources, categorizing food outlets, standardizing field observations and validation protocols, and reporting over and under count errors. The US National Collaborative on Childhood Obesity Research (http://www.nccor.org) could compile these common metrics on their online database. Given the reliability and field’s reliance on local health data, researchers may want to work with local, regional, tribal, or state offices to improve the collection and archiving of local food environment data [50,51].

## Conclusions

Public health professionals need to consider the validity of local food environment data whether advocating, developing, implementing, or evaluating environmental and policy strategies to improve access to healthy, affordable foods. ReferenceUSA and local health department data provided a relatively accurate identification of the local food environment in American Indian communities in NC. However, secondary data sources over and under counted the food outlets and were particularly problematic for identifying convenience stores and specialty markets. More efforts are needed to improve the validity of existing data sources, especially in rural areas.

## Abbreviations

CDP: Census-Designated Place; GIS: Geographical Information Systems; GPS: Global Positioning Systems; NC: North Carolina; NEMS: Nutrition Environment Measurement Survey; NIH: National Institutes of Health; RUCA: Rural–Urban Commuting Area; RWJF: Robert Wood Johnson Foundation; SDTSA: State Designated Tribal Statistical Area; US: United States; USDA: United States Department of Agriculture; WIC: Women, Infants, and Children.

## Competing interests

The authors declare that they have no competing interests.

## Authors’ contributions

All authors read and approved the final manuscript. Dr. SEF led the conception and design, acquisition of data, along with analysis and interpretation of the data. Dr. DAR contributed to the conception and design, along with analysis and interpretation of the data. Dr. KRE contributed to the design, specifically ground-truthing protocol, along with analysis and interpretation of the data. Mrs. AH contributed to acquisition of ReferenceUSA and Dun & Bradstreet data and spatial analyses. Dr. ZG contributed to the statistical analysis and interpretation of statistical data. Ms. DS assisted with inter-rater reliability data collection and analysis, along with geocoding and food outlet editing. And, Ms. GR contributed to online yellow page data collection, categorization of food outlets, and food outlet editing.

## Supplementary Material

Additional file 1 Condensed protocols for six data sources and approaches used to collect information on a wide range of food outlets in six State Designated Tribal Statistical Areas and two US Census-Designated Places in North Carolina, 2009-2010. Click here for file

Additional file 2 Ground-truthing Protocol. Click here for file
