# Supplementary material for: Evidence for validity of five secondary data sources for enumerating retail food outlets in seven American Indian Communities in North Carolina
Source: Int J Behav Nutr Phys Act. 2012 Nov 22;9:137. doi: 10.1186/1479-5868-9-137 (PMC3551728; doi:10.1186/1479-5868-9-137)
Supplement: Additional file 1 — Condensed protocols for six data sources and approaches used to collect information on a wide range of food outlets in six State Designated Tribal Statistical Areas and two US Census-Designated Places in North Carolina, 2009-2010. [file 1479-5868-9-137-S1.doc]

Condensed protocols for six data sources and approaches used to collect information on a wide range of food outlets in six State Designated Tribal Statistical Areas and two US Census-Designated Places in North Carolina, 2009-2010

| **Data Source Selection Rationale & General Instructions** | **Inclusion & Exclusion Protocol** |
| --- | --- |
| **Online Yellow Pages** | |
| *Rationale for Use*: Online Yellow Pages ([http://www.yellowpages.com](http://www.yellowpages.com/)) provides easy, free, and quickly accessible information on food sources by location. In a recent review, 13 out of 40 of the fast food access studies relied on online telephone directories such as the Yellow Pages.[1]  *Instructions*: “Food” was entered into the search box labeled “find” for each ZIP Code co-locating with each State-Designated Tribal Statistical Area (SDTSA). Only outlets physically located within our ZIP Code of interest were included.  *Protocol Development Process*: Multiple researchers (SF, AR, KE, DR) reviewed the protocol. One researcher (SF) piloted the protocol using ZIP Codes originally co-locating with one SDTSA in 2000 but not co-locating after 2010 modifications to the SDTSA. Two additional researchers (GR, AR) helped refine the protocol, using it to gather information on some of our more rural ZIP Codes. Our final protocol was detailed enough to consistently click on the same words, images, or icons. During data collection, Internet searches and phone calls were made to try to ascertain all necessary information for any incomplete listing because a number of food outlets had missing names, addresses, or ZIP Codes. If it was questionable whether an outlet served food to the public (e.g., 3 R’s Auto Service), the outlet was called to verify if food is available at the outlet and the outlet is publicly accessible (e.g., not a privately owned country club). | *Included*: canners & food processors, convenience stores, fast food restaurants, food and beverage consultants, food banks, food delivery service, food facilities consultants, food processing and manufacturing, food processing equipment and supplies, food products, food products-wholesale, food service management, frozen food locker plants, frozen food, frozen food-wholesale, fruit and vegetable-wholesale, fruit and vegetable markets, grocers-ethnic foods, grocers-specialty foods, grocers-wholesale, grocery stores, health and diet food products, health and diet food products-wholesale, health food restaurants, Mexican food products, natural food, nuts-edible, restaurants, soul food restaurants, and vitamins and food supplements  *Excluded*: auto repair shop, banquet hall, barbeque sauce vendor, bingo hall, bottling company, bowling alley, can manufacturing company, catering, children’s clothing store, food distributor, edible arrangements, exercise consultant, commercial farming company, feed store, food service management company, gift basket store, hardware stores, home health club, hotel restaurant, indoor playgrounds, liquor stores, massage therapy, nightclubs, military products, nutrition consultant, packaging, pet supply store, private golf country club, processing plant, restaurant supply store, roller ring, RV Park, talent search agency, tax preparation services, tile imports, timber, used car store, used furniture store, variety store, vending company, video market, vitamin supplement store, water distributor, and wigs |
| **Local Health County Food Inspection Listings** | |
| *Rationale for Use*: North Carolina Division of Environmental Health authorizes local counties to review food service plans, permit and inspect certain food venues, and lead investigations into food borne illnesses and general complaints regarding food service establishments. County health department food inspections lists are public government information, generally accessible to the public for free or a low-cost.  *Instructions*: All co-locating North Carolina counties (n=21) were called in fall 2009. The North Carolina Division Environmental Health compiles all local county data but we opted to consistently call and collect our data from the local level in hopes it would produce the most up to date listing and help connect us with the person with most knowledge of the local food environment in the selected counties. All 21 counties mailed, emailed, or faxed free copies of their latest inspection lists or directed us to a website where their local food inspection data could be accessed and downloaded for free via the Internet.  *Protocol Development Process*: Multiple researchers (SF, AR, KE, DR) reviewed the protocol and one researcher (SF) collected and entered all the local health county data. SF also compared a couple local county listings from urban and rural areas to information gathered by the State and found minor differences between the local and State listing. Local inspectors were called a number of times to help identify non-food and closed outlets. Before talking with the local inspectors, SF and GR tried to call and research via the Internet to establish whether or not questionable outlets regularly served food to the public. | *Included*: food stands, meat markets, mobile food units, pushcarts, and restaurants  *Considered*: bed and breakfast homes/inns, country clubs, and lodging (called and inquired if publicly accessible; majority were not publicly accessible)  *Excluded*: daycares, private or public school lunchrooms and boarding schools, college food service venues, educational food service, elderly nutrition sites, summer camps, primitive camp, resident camps, pools, spas, hospitals, medical centers, nursing homes, residential homes, institutional food service (e.g., prisons), worksite cafeterias, adult day care, tattoo artists, temporary concessions or limited food service (e.g., high school concession stands or carnival/fair concession stands), movie theaters, nightclubs, sport or activity centers (e.g., soccer centers or ice skating rings), and bowling alleys |
| **State Department of Agriculture and Consumer Services Food Inspection Listings** | |
| *Rationale for Use*: North Carolina authorizes the State Department of Agriculture and Consumer Services to enforce state and federal food safety laws, particularly relating to meat and poultry inspection, meat processing plants, farm slaughter activities, processing and marketing of certain meats, farmers’ markets, canneries, and bakeries. The State Department’s list is public government information, generally accessible to the public for free or a low-cost.  *Instructions*: The State Department provided us with an up-to-date listing of all food establishments it inspects within all co-locating North Carolina counties (n=21) in December 2009.  *Protocol Development Process*: Multiple researchers (SF, KE, DR) reviewed the protocol and one researcher (SF) collected and entered all the data. Calls were made or emails were sent to the State Department if an outlet had incomplete information or we had a question about the types of food an outlet sold or if an outlet sold food publicly. | *Included*: bakeries, farmers’ markets, and stores with packaged goods sold to the public  *Excluded*: canning, slaughtering, or processing facilities that did not sell food publicly |
| **ReferenceUSA** | |
| *Rationale for Use*: Frequently described in the literature as one of the most comprehensive commercial data sources available, ReferenceUSA categorizes food types using the North American Industry Classification System (NAICS) and provides latitude and longitude data. NAICS is the standard used by the Federal government to classify and examine business establishments.  *Instructions*: Using our university’s e-research tools, we accessed ReferenceUSA. We conducted a custom search for our selected NAICS codes found within all co-locating North Carolina ZIP Codes (n=78). We gathered all NAICS outlets by ZIP Code. The outlets identified through this search were reviewed and sorted to eliminate or flag any potential questionable food outlets or delete duplicates.  *Protocol Development Process*: Multiple researchers (SF, AH, KE, DR) reviewed the protocol and one researcher (SF) collected and entered all the data. To ensure consistency in data protocol, SF compared the search strategy with search strategies and outputs obtained from two different trial runs conducted with two different reference librarians. Calls were made to all pharmacy outlets identified to ensure food was sold at each location. | *Included*:  445 Food and Beverage Sales  4451 Grocery Stores  445110 Supermarkets and Other Grocery (except Convenience) Stores  445120 Convenience Stores  4452 Specialty Food Stores  445210 Meat Markets  445220 Fish and Seafood Markets  445230 Fruit and Vegetable Markets  445291 Baked Goods Stores  445292 Confectionery and Nut Stores  445299 All Other Specialty Food Stores  447 Gasoline Stations  447110 Gasoline Stations with Convenience Stores  72 Accommodation and Food Services  722 Food Service and Drinking Places  7221 Full-Service Restaurants  722110 Full Service Restaurants  7222 Limited-Service Eating Places  722211 Limited-Service Restaurants  722212 Cafeteria, Grills Buffets, and Buffets  722213 Snack and Nonalcoholic Beverage Bars  4299 Other General Merchandise Stores  452910 Warehouse Clubs and Superstores  452990 All Other General Merchandise Stores  452112 Discounted Department Stores  446110 Pharmacies and Drug Stores  *Excluded*: appliance store, car wash, clothing store, concrete services, distribution, feed store, food supplies, home store, hotel, medical equipment, pharmacy, vegetable grower, and water supplier and distributor |
| **Dun & Bradstreet** | |
| *Rationale for Use*: Frequently described in the literature as one of the most comprehensive commercial data sources available, Dun & Bradstreet categorizes food types using the North American Industry Classification System (NAICS) and provides latitude and longitude of the outlet.  *Instructions*: Using resources from the North Carolina Department of Commerce, Economic Development Intelligence Systems, we accessed without charge Dun & Bradstreet. We conducted a custom search for our selected NAICS codes found within all co-locating North Carolina counties (n=21). We gathered all NAICS outlets by county. The outlets identified through this search were reviewed and sorted to eliminate or flag any potential questionable food outlets or delete duplicates.  *Protocol Development Process*: Multiple researchers (SF, AH, KE, DR) reviewed the protocol and one researcher (SF) collected and entered all the data. To ensure consistency in data protocol, SF compared her search strategy and final run outcomes with search strategies and outputs obtained from one trial run conducted with a reference librarian. Calls were generally made to all pharmacy outlets identified to ensure food was sold at each location. | *Included*: Same NAICS included in ReferenceUSA.  *Excluded*: athletic facility, clothing store, concrete services, electronic store, home store, medical equipment, pharmacy, and school |
| **Ground-truthing, Global Positioning Systems Assisted** | |
| *Rationale for Use*: Ground-truthing is recommended as the most valid and reliable way to examine the food environment, particularly in rural settings.[2] Ground-truthing uses trained observers to directly and systematically canvass the study area with or without Global Positioning Systems (GPS) and without a list of food outlets to guide the canvass. Ground-truthing has been considered the “gold standard” and recommended over secondary data sources since studies had shown secondary lists when compared with ground-truthing both under- and over-estimated food access.  *Instructions*: Using a modified version of Dr. Joseph Sharkey’s ground-truthing protocol[3, 4], we drove all 2009 TIGER/Line primary, secondary, and local roads within our SDTSA and determined the location (latitude and longitude) of all food outlets identified within or just outside our SDTSA. Specifically, all lines were downloaded for counties co-located with the seven SDTSAs and subset to show only roads features. Each line has a Feature Classification Code identifying what type of feature it depicts.  The following MAF/TIGER Feature Class Codes (MTFCC) was used: primary road (S1100), secondary road (S1200), and local neighbourhood road, rural road, city street (S1400). Once the features were subset for each county, the data was merged to form a single shapefile, and then it was projected into the North Carolina State Plane (meters) coordinate system.  The length of each line was then calculated and stored as an attribute in the roads attribute table.  The identity tool was used to assign all of the attributes from the SDTSAs to the road features they intersect.  Finally, the summarize tool was used to sum all of the line lengths by SDTSA name.  We toured some of our initial tribes using a variety of online mapping tools to get a sense of our areas, including Google Street Maps, Google Earth, and the ArcGIS Online web mapping services World Imagery, DeLorme, and Topographic. For our more urban tribe, we focused on ground-truthing two US Census Places. Tribal liaisons were asked to review their respective tribe’s map(s) before or during the food assessments and encouraged to provide any input on the maps or particular key food outlets missed if we only stayed on the printed path.  *Protocol Development Process*: Multiple researchers (SF, KE, DR, AH, AR) reviewed the protocol.  *Pilots:* A series of pilots were conducted in Cary, Chapel Hill, Durham, and Pittsboro, North Carolina, over 100 miles canvassed. In Cary, we piloted a windshield survey approach on 38 outlets[3] and on a different ground-truthing survey, we piloted another protocol of Dr. Joseph Sharkey[3, 4] on 25 outlets in Cary. Our final protocol was piloted in Chapel Hill, Durham, and Pittsboro on 25 outlets.  *Inter-rater reliability:* We conducted an inter-rater reliability process of 10% of all of our roads within the SDTSA for six of the tribes and 10% of all roads within the more urban of the 2 Census-Designated Places examined and had high agreement. | *Included*: Any outlet open, regularly selling foods and/or beverages to the public. The outlet needed to have at least one shelf or rack of food. An outlet or area with only vending machines were noted but were not considered a food outlet.  *Excluded*: access-restricted buildings, such as schools, hospitals, or office buildings, bowling alley, institutional food service operations, strip clubs, roller ring, and temporary food establishment |

1. Fleischhacker S, Evenson K, Rodriguez D, Ammerman A: **A systematic review of fast food access studies** *Obes Rev* 2010, **12:**e460-e471.

2. Sharkey J: **Measuring potential access to food stores and food-service places in rural areas in the US.** *Am J Prev Med* 2009, **36:**S151-S155.

3. Sharkey J, Horel S: **Neighborhood socioeconomic deprivation and minority composition are associated with better potential spatial access to the food environment in a large rural area.** *J Nutr* 2008, **138:**620-627.

4. Sharkey J, Horel S, Han D, Huber J: **Association between neighborhood need and spatial access to food stores and fast food restaurants in neighborhoods of *colonias*.** *Int J Health Geographics* 2009, **8**.
