# Supplementary material for: Evidence for validity of five secondary data sources for enumerating retail food outlets in seven American Indian Communities in North Carolina
Source: Int J Behav Nutr Phys Act. 2012 Nov 22;9:137. doi: 10.1186/1479-5868-9-137 (PMC3551728; doi:10.1186/1479-5868-9-137)
Supplement: Additional file 2 — Ground-truthing Protocol. [file 1479-5868-9-137-S2.doc]

**Ground-truthing Protocol**

BEFORE YOU HIT THE ROAD

Each tribe will have its own ground-truthing packet, including the necessary maps, comprehensive food list, letters/flyers for possible folks encountered along the way, and blank outlet observation sheets to record all outlets identified and surveyed during the ground-truth process. If possible, [names] will try to call each tribe’s tribal administrator or executive director and see if the team can visit with him/her while cataloging the tribe’s food outlets. Please make sure you review the following checklist before you leave:

CHECKLIST

- Tribe packet/binder with:
  - Maps
  - Comprehensive food outlets list
  - Copies of letters/flyers to distribute
  - Mileage log and outlet observation sheets
  - Directions to get to and from the tribe
  - Hotel and food recommendations
- GPS unit (can save about a 1,000 outlets)
  - Charged
  - Adapter for computer charging and downloading
  - Car charger
  - GPS container
  - Car GPS stand
- Camera
  - Charged
  - Charger
  - Adapter for computer downloading
- Reserve university vehicle and ensure gas card is in car and GPS unit works
  - Each team member must have an agreement and copy of their Driver’s License on file
- Snacks/water for the car and music for the commute
- Emergency contact numbers for university and each team member

MAPS

1. Use the following maps to first visually examine the route to be driven:
   1. TIGER roads 2009
   2. World Imagery
   3. DeLorme
   4. Topo
   5. Google Street Maps
   6. Google Earth
   7. GIS-map of our comprehensive intermediate food outlet data
2. Circulate tribe map link(s) and listing to planning team to indicate roads considering deselecting, including apartment, condos, and trailer park roads. You may deselect these types of roads if you see them during your drive and they had not been originally deselected in your maps.
3. If possible, pick up a local county map at a nearby Walgreen’s or Gas Station when you arrive in town. Compare this map with the above maps.

BEFORE YOU START DRIVING YOUR AREA

1. At least two trained team members are required to ground-truth at one time.
2. Ground-truthing should take place during daylight hours and/or normal business hours (9 am to 5 pm).
3. We will track total mileage driven for each tribe’s ground-truthing. We will calculate total miles driven from the university to the tribe, within the tribal area for ground-truthing per day, and miles driven back home to the university. This information will help us understand how long it takes to ground-truth and also help us stay within our travel budget. Please complete the following log sheet at the beginning and end of your drive. In addition, once you reach your designated tribal roads record per day the starting and ending mileage driven by day.

**Tribe Traveling Log Sheet**

Team:_____________________________________________________________________________________________________________________________________________________

Date drove to tribe:______________________________________________________________

Starting Mileage (Odometer reading):_______________________________________________

*Complete a daily log, recording daily mileage and route driven*

**Day One**

Day One Date (Date of Week, Month Day, Year):______________________________________

Day One Starting Odometer Reading (does not include the miles you drove to get to the tribe, it is the Odometer Reading once you get to the first road listed on your Google Street Map to ground truth):__________________________________________________________________

Driving Start Time:_____________________________________________________________

Break Periods:__________________________________________________________________

Driving End Time :______________________________________________________________

Roads/Route Driven (name the roads you drove, referring to the Google Street’s Map):________

__________________________________________________________________________________________________________________________________________________________________________________________________________________________________________

Day One Ending Odometer Mileage (take this reading once you stop ground-truthing, not when you reach, for instance, where you’ll eat dinner or stay the night):_________________________

**Day Two**

Day Two Date (Date of Week, Month Day, Year):_____________________________________

Day Two Starting Odometer Reading (does not include the miles you drove to get to the tribe, it is the Odometer Reading once you get to the first road listed on your Google Street Map to ground truth):__________________________________________________________________

Driving Start Time:_____________________________________________________________

Break Periods:__________________________________________________________________

Driving End Time :______________________________________________________________

Roads/Route Driven (name the roads you drove, referring to the Google Street’s Map):________

__________________________________________________________________________________________________________________________________________________________________________________________________________________________________________

Day Two Ending Odometer Mileage (take this reading once you stop ground-truthing, not when you reach, for instance, where you’ll eat dinner or stay the night):_________________________

**Day Three**

Day Three Date (Date of Week, Month Day, Year):____________________________________

Day Three Starting Odometer Reading (does not include the miles you drove to get to the tribe, it is the Odometer Reading once you get to the first road listed on your Google Street Map to ground truth):__________________________________________________________________

Driving Start Time:_____________________________________________________________

Break Periods:__________________________________________________________________

Driving End Time :______________________________________________________________

Roads/Route Driven (name the roads you drove, referring to the Google Street’s Map):________

__________________________________________________________________________________________________________________________________________________________________________________________________________________________________________

Day Three Ending Odometer Mileage (take this reading once you stop ground-truthing, not when you reach, for instance, where you’ll eat dinner or stay the night):_________________________

**Day Four**

Day Four Date (Date of Week, Month Day, Year):_____________________________________

Day Four Starting Odometer Reading (does not include the miles you drove to get to the tribe, it is the Odometer Reading once you get to the first road listed on your Google Street Map to ground truth):__________________________________________________________________

Driving Start Time:_____________________________________________________________

Break Periods:__________________________________________________________________

Driving End Time :______________________________________________________________

Roads/Route Driven (name the roads you drove, referring to the Google Street’s Map):________

__________________________________________________________________________________________________________________________________________________________________________________________________________________________________________

Day Four Ending Odometer Mileage (take this reading once you stop ground-truthing, not when you reach, for instance, where you’ll eat dinner or stay the night):_________________________

**Day Five**

Day Five Date (Date of Week, Month Day, Year):_____________________________________

Day Five Starting Odometer Reading (does not include the miles you drove to get to the tribe, it is the Odometer Reading once you get to the first road listed on your Google Street Map to ground truth):__________________________________________________________________

Driving Start Time:_____________________________________________________________

Break Periods:__________________________________________________________________

Driving End Time :______________________________________________________________

Roads/Route Driven (name the roads you drove, referring to the Google Street’s Map):________

__________________________________________________________________________________________________________________________________________________________________________________________________________________________________________

Day Five Ending Odometer Mileage (take this reading once you stop ground-truthing, not when you reach, for instance, where you’ll eat dinner or stay the night):_________________________

**Day Six**

Day Six Date (Date of Week, Month Day, Year):______________________________________

Day Six Starting Odometer Reading (does not include the miles you drove to get to the tribe, it is the Odometer Reading once you get to the first road listed on your Google Street Map to ground truth):__________________________________________________________________

Driving Start Time:_____________________________________________________________

Break Periods:__________________________________________________________________

Driving End Time :______________________________________________________________

Roads/Route Driven (name the roads you drove, referring to the Google Street’s Map):________

__________________________________________________________________________________________________________________________________________________________________________________________________________________________________________

Day Six Ending Odometer Mileage (take this reading once you stop ground-truthing, not when you reach, for instance, where you’ll eat dinner or stay the night):_________________________

**Day Seven**

Day Seven Date (Date of Week, Month Day, Year):____________________________________

Day Seven Starting Odometer Reading (does not include the miles you drove to get to the tribe, it is the Odometer Reading once you get to the first road listed on your Google Street Map to ground truth):__________________________________________________________________

Driving Start Time:_____________________________________________________________

Break Periods:__________________________________________________________________

Driving End Time :______________________________________________________________

Roads/Route Driven (name the roads you drove, referring to the Google Street’s Map):________

__________________________________________________________________________________________________________________________________________________________________________________________________________________________________________

Day Seven Ending Odometer Mileage (take this reading once you stop ground-truthing, not when you reach, for instance, where you’ll eat dinner or stay the night):____________________

SPOTTING OUTLETS

1. As you begin driving your route, the passenger(s) should be focused on spotting any food outlets seen.
2. When an outlet is located, the driver should carefully pull over to the outlet and park in a safe spot. Ideally, position the car in front of the food outlet. If you cannot take a GPS reading close to the food outlet, carefully pop the GPS unit out of its car holder and carry the unit with you and take a GPS lat/long while you physically stand in front of the food outlet. Take the lat/long of each food outlet by asking the car GPS: Where am I? The GPS Unit will display the location’s lat/long. Save the coordinates by selecting next, then type in the name of the food outlet (e.g., McDonald’s). And, then go back to coordinates to be ready to take the next outlet’s latitude/longitude.
3. If you leave the car, make sure the car is locked, the GPS unit is packed up and stored in the trunk, and the letter below (on page 8) is put on the dashboard of the car.

ENCOUNTERING PEOPLE WHILE GROUND-TRUTHING

1. Provide the below letter (page 8) to any folks you encounter throughout the ground-truth process. Please call [PI] at [phone number], if you encounter any problems. Each team will be provided with multiple copies of this letter, and possibly flyers to recruit key informants.

TAKING PICTURES OF FOOD OUTLETS

1. Make sure the flash is off and take a picture of the lat/long provided on the GPS Unit.
2. Take at least one photo of the food outlet, capturing the name of the outlet. Take any additional photos that may help describe the physical location of the outlet (e.g., in a mall area or in K-Mart) or the type of outlet (e.g., full-service restaurant with to-go option).

ON UNIVERSITY LETTERHEAD

January 24, 2010

Hello,

During the winter and spring of 2010, we will be visiting seven North Carolina tribal communities: Coharie Tribe, Haliwa Saponi Indian Tribe, Lumbee Tribe, Meherrin Indian Tribe, Occaneechi Band of Saponi Nation, Sappony, and Waccamaw Siouan Tribe.

We will be exploring the types of food outlets within each of these communities to help us develop planning and policy strategies to improve access to healthy eating within North Carolina American Indian communities.

Research teams will be driving all the major roads within each of the seven participating tribal communities at least once, possibly twice, in order to collect the physical location of each food outlet and some basic information on the type of food outlet (e.g., opened/closed and name of location).

This environmental assessment is part of a larger healthy eating project we are working on with the North Carolina Commission of Indian Affairs and the seven participating North Carolina tribes. This project was approved by the [name of university] Institutional Review Board. We do not work for the city or state government. We will present our findings to each tribe sometime late summer/early fall.

If you have any questions about this study, please call me at [phone number]. Thank you for your interest in our project.

Sincerely,

OUTLET OBSERVATIONS

1. After the lat/long has been recorded in the GPS unit and at least one photograph of the outlet has been taken, complete the outlet observation below. Keep the outlet observations aligned with your GPS recordings and photographs using the pre-assigned **Site ID** (tribe name_number in order of GPS lat/long recording).
2. Document the **food outlet name**. If an outlet is closed or the outlet space is vacant, indicate what was their previously and note the store is closed. If an outlet is opening, indicate the outlet name and that it is opening soon. If possible, note the opening date. Describe the physical layout of the food outlet. A **fixed** outlet is a permanent structure while a **mobile** outlet is mobile, such as a food truck.
3. If fixed, circle one of the following to further describe the physical layout: stand alone, attached, or nested-within.  **Stand Alone** is an outlet with no other store(s) attached to it.  **Attached** means, for instance, Subway shares a wall with a Hair Palor. For attached outlets, list the name of the store(s) the food outlet is attached to (e.g., Sara’s Hair) in the line next to attached term. An outlet may or may not be attached to another outlet on both its left and right sides. **Nested-within** means, for instance, Subway is nested within Wal-Mart. For nested-within outlets, list the name of the larger store the food outlet is nested within (e.g., Wal-Mart) in the line next to the nested-within term.
4. An outlet can be **same-lot** in addition to stand alone, attached, or nested-within. Same-Lot means, for instance, Subway shares a parking lot with McDonald’s, Target, and Borders. For same-lot outlets, list the name of the stores sharing the same lot provided next to same-lot term. If needed, use the bottom or back of the page to complete the listing of outlets. General names such as “dry cleaning” work for non-food outlets. For any subsequent outlet observations for outlets within this same lot, you do not have to complete this listing per outlet. Please just circle same-lot for any subsequent outlets in a same-lot and note the Site ID for the first outlet in the same-lot you listed all the outlets within the same-lot. If you are starting to record outlets in a same-lot situation, you can opt to take the previous food outlet observation sheets out of the tribe binder and store in the tribe ground truth file binder. This may make it easier to keep outlets within a same-lot situation grouped together.
5. **Food Court/Food Options inside a Mall** means the outlet is within a mall where you have to go inside the mall to primarily access the food outlet. For food court/food options inside a mall, list the name of all food outlets within the food court/mall area, as best as possible. Use the mall directory as a guide to help you find all the food outlets within the mall. If needed, use the bottom or back of the page to complete the listing of outlets. For any subsequent outlet observations for outlets within this food court/mall, you do not have to complete this listing per outlet. Please just circle the food court/food options for any subsequent outlets in a food court/mall and note the Site ID for the first outlet in the food court you listed all the outlets within the food court/mall.
6. **Nutrition Environment Measures Survey Outlet Classification^1, 2^**. Use the below modified NEMS store and restaurant classification codes to classify the type of outlet you are observing. The list of codes will be in the tribe’s ground-truth binder front/back insert. The following outlet classifications and corresponding codes are based on the Nutrition Environment Measures Survey Restaurant and Stores Survey with slight adjustments to accommodate a systematically numbering scheme between stores and restaurants and to accommodate a broader list of food outlets.

For instance, list Sam’s Club classification code as S01. If you cannot find an appropriate NEMS classification code, indicate other (S13 or R27) and provide as much information as possible regarding this type of outlet (e.g., coffee store within a book store).

***Stores***

S00: Store, cannot determine specifically its type (to use for intermediate data classifications)

S01: Member-Only Supercenter (e.g., Cosco or Sam’s Club)

S02: Supercenter (e.g., Super Target and Walmart)

S03: Grocery Store (e.g., Harris Teeter or Lowes)

S04: Convenience Store (e.g., Corner Store or Small General Stores or Bait and Grocery Shops, includes convenience stores with grill offerings)

S05: Convenience Store with Gas Station (e.g., BP Station, includes convenience stores with grill offerings)

S06: Mass Merchant (e.g., Big Lots) and Variety or General Retailer (includes Kmart and Walmart that are not Super with food)

S07: Dollar Store (e.g., Maxway)

S08: Pharmacy (e.g., CVS). Note some pharmacy have less food than CVS, Walgreen’s, and Rite Aid. Please note if you identify a non-chain pharmacy the type and extent of food and beverage offerings within the store or vending just outside the store.

S09: Alcoholic Beverage Store with Food (e.g., Total Wine)

S10: Specialty Market, including meat (e.g., butcher), fish, fruit/vegetable, cheese, ethic, gift & gourmet store, health foods

S11: Flea Market, including food mobile units

S12: Farmers’ Market or Public Farm Products, includes picking produce

S13: Food Bank or Soup Kitchen

S14: Other

S15: Smaller Grocery Store

***Restaurants***

R00: Restaurant, cannot determine specifically its type (to use for intermediate data classifications)

R01: General/Mixed/American (e.g., Applebees, Chili’s, TGIFriday, IHOP, and Waffle House)

R02: Burgers (e.g., McDonald’s, Wendy’s, Hardees)

R03: Chicken (e.g., KFC, Popeyes, Church’s Chicken, Chick-Fil-A, Bojangles)

R04: Sandwiches (e.g., Subway, Blimpie, Quiznos, Arby’s, Café-Style Delis, such as Panera Bread)

R05: Pizza (e.g., Pizza Inn, Pizza Hut, Mellow Mushroom)

R06: Bagels Plus (e.g., Einstein’s, Bagel Palace Deli)

R07: Biscuits (e.g., Biscuitville)

R08: Donut Shops (e.g., Krispy Kreme, Dunkin Donuts)

R09: Bakery/Pastry Shops/Sweets (e.g., Mrs. Field’s Cookies, Cinnabon, Pretzel Shops)

R10: Coffee or Tea Outlets Plus (e.g., Starbucks, Seattle’s Best Coffee, and Caribou Coffee)

R11: Ice Cream/Frozen Yogurt Shops/Smoothies (e.g., Baskin Robbins, TCBY, Cold Stone Creamery, Baskin Robbins, and Java Juice)

R12: Seafood (e.g., Red Lobster)

R13: BBQ (e.g., Smithfield’s)

R14: Steakhouse (e.g., Outback Steakhouse, Longhorn)

R15: Bars/Pubs (e.g., ESPN Zone) Wine

R16: Asian (mixed, other including Korean) (e.g., Mama Fu’s, Chopsticks, Top Spice)

R17: Chinese (e.g., Red Pepper, The Golden Buddha, and Panda Chinese)

R18: Thai (e.g., Thai Chili, Thaicoon)

R19: Japanese (e.g., Edo Steak House, Shogun, Fuji Japanese)

R20: Mexican (e.g., Taco Bell, Chipotle, Moe’s, Qdoba, Pappasitos, Don Pablos)

R21: Italian (e.g., Sbarro, Olive Garden, Romano’s Macaroni Grill)

R22: French (e.g., Pettie Auberge, Le Madeline)

R23; Indian (e.g., Touch of India, Himalayas Indian, Haveli)

R24: Greek, Middle Eastern (e.g., Athens Restaurant, Kyma, Basil’s Mediterranean)

R25: Soul Food

R26: Ethiopian

R27: Vegetarian

R28: Spanish (e.g., Tapas)

R29: Hot Dog Stand

R30: Other

1. **For restaurants**, please use one or more of the following options to describe the type of service provided:

**Fast Food Restaurant** (e.g., limited service, counter-only, McDonald’s)

**Fast-Casual Restaurant** (e.g., order at counter, but delivered to your table, Corner Bakery); **Full-Service Restaurant** (waiter comes to your table and takes your order);

**Buffet-Style Restaurant** (all you can eat buffet option);

**Banquet** (e.g., weddings, special events);

**Catering** (e.g., bring food to you);

**Delivery** (pizza); and

**To-Go or Drive-Thru** (pick up and go).

**Outlet Observation Site ID** (Tribe’s Name_Number in ascending order of review):

**Food Outlet Name:____________________________________________________________________**

**Physical Layout** (Circle): Fixed or Mobile Then, Circle/Complete:

Stand Alone

Attached to____________________________________________________________________

Nested-within__________________________________________________________________

Same-lot outlets (or Site ID for first):________________________________________________

_________________________________________________________________________________________________________________________________________________________________________________________________________________________________________________________________________________________________________________________________________________________________________________________________________________________________________

Food Court/Food Options inside a Mall (or Site ID for first):_____________________________

_________________________________________________________________________________________________________________________________________________________________________________________________________________________________________________________________________________________________________________________________________________________________________________________________________________________________________

**Nutrition Environment Measures Survey Outlet Classification**:_________ If other, describe outlet further___________________________________________________________________

**For restaurants**, please Circle one or more of the options below to describe the type of service at the restaurant: Fast Food Restaurant; Fast-Casual Restaurant; Full-Service Restaurant; Buffet-Style Restaurant; Banquet; Catering; Delivery; and To-Go or Drive-Thru

DOWNLOADING GPS & CAMERA AFTER EACH DAY OF GROUND-TRUTHING

Download your day’s work to the project computer (i.e., PI’s labtop). These files are backed up on PI’s Mozy account. You should download the files from GPS and pictures from the camera as indicated below. Then, make sure to charge the GPS by leaving it plugged into the computer and the camera battery plugged into the wall charger. Make sure to not leave any of the instruments or chargers behind at the hotel!

GPS: Use the GPS UPS cord to plug in GPS to computer. Turn GPS on by hitting the power switch. Go to my computer on the project computer and click on the Garmin icon. Open the

GPX folder and select current and right click to move to GPS Data folder in RWJF NCAI Food Access Project folder. Rename current GPX file to 2010monthdate_tribe (insert ground-truthing date and specific tribe name). Go to remove hardware safely. Disconnect, tools, scroll down, my data, delete favorites, select all, delete, turn power off.

Google Earth: Click Google Earth icon, drag and drop your GPS file from RWJF NCAI Food Access project folder into Google Earth. Check all Google Earth pop-up options and click okay. Move from temporary files to my places, drag and drop. Delete bad outlets. Delete tracks. Rename any misspelled outlets. Name if needed any coordinates not named. Right click on data file and save to project folder. If needed to improve placement of an outlet’s lat/log in Google Earth, click yellow thumb tack and move it to where you want the outlet way point. Then, cut and paste the lat/long into the description box and copy and close. To save, right click on folder save places as, same file name.

Camera: Turn on camera and use the camera UPS plug or the GPS UPS plug to attach the camera to the project computer. The camera should automatically download the pictures into Picasa. Select all the pictures of that ground-truthing day. The pictures should import into the project folder, entitled RWJF NCAI Food Access Project. Add a new folder to the project folder using the following convention: 2010monthday_tribe name; for instance, 20100210_Lumbee. After copying pictures from camera to computer, delete only copied photos and remove imported files. Remove the safely the hardware from the computer and then turn off the camera and charge the battery using the wall charger. In Picasa, make captions for each photo using the name of the tribe, date of ground-truthing, and then the outlet’s name, lat/long, and why you think this photo is helpful, such as indicating outdoor sitting. Ensure each image is named using the date_tribe convention plus the name of the outlet. The outlets should be in order of their lat/long, lining up with the GPS file. You can insert these photographs into GPS, if time permits on the road.

BEFORE RETURNING THE CAR TO UNC

1. Ensure your mileage log listed above is completed, including your final mileage reading when you arrive back at UNC.
2. Make sure the University car has at least a half of tank of gas and the keys are returned to Renee or Motor CarPool.

**Food Outlet Classification-Modified**

**from the Trial of Activity for Adolescent Girls Food (TAAG) Ancillary Study**

The type of food outlets for the TAAG ancillary study are defined as follows:

1.Convenience Stores (type 1, 7)
Convenience stores/food marts are primarily engaged in retailing a limited line of goods that generally includes milk, bread, soda, and snacks.  These establishments are primarily engaged in retailing automotive fuels in combination with a convenience store or food mart. (Might have the following in the name: Quick, Quik, Mini, Mart).  This also includes liquor stores, gas stations, and pharmacies (type 7) that sell food. 1 includes our NEMS S04, S05, S15, S06, S07, S08, S09.

2. Specialty Market (type 14)
Sells just one type of food, mainly for consumption and/or preparation at home (e.g., Meat (butcher), Seafood/Fish, Poultry, Cheese, Wine/winery, Water/Beverages).

3. Markets/Produce Stores (type 2)
Markets and produce stores (also known as bodegas) is defined as a small supermarket.  Their primary line of retail is food, but they are typically not chain establishments and have more limited offerings than supermarkets.  A produce stand also counts here. Added Flea Market, since the food vendors in our study tended to all be selling preliminary produce items.

4. Grocery Stores/ Supermarket (type 3)
Larger than markets/produce stores, often chain establishments, have a wide variety of products.  Grocery stores/supermarket have several cash registers for check-out—usually larger than 5000 sq feet.   Trader Joes and larger would be a grocery store/supermarket.

5. Warehouse Stores (type 11)
Larger than supermarkets, offer large quantities or higher discounts than other stores.  Includes: Sam’s Club, Costco, Commissary, Wal-Mart.

Prepared Food Outlets

6. Fast Food (type 4)
Fast food is characterized by minimal service, fast preparation, and ordering at the counter or drive through. Fast food establishments do not have wait staff. Restaurants located in a food court or limited to take-out only are considered fast food.

7. Sit-down Restaurant, Buffets, Cafeterias, and Fast-Casual Restaurants (type 5, 45)
Sit-down restaurants are characterized by establishments that offer table service with wait staff who take your order at the table, or where food can be chosen from a buffet or cafeteria style.  Majority of business is sit in and take out is a smaller fraction.  For fast casual (type 45), food is ordered or paid for at the counter but delivered to the table.

8. Coffee Shops (type 27)
Coffee/tea are the main items for sale, on site consumption or to go. Includes key words in the business name: Coffee, Starbucks, Java, Bean, Perk, Grind, Espresso/Expresso, Brew, Tea.

9. Specialty Prepared Food Shops (type 6, 28)
Limited type of food product available ready to eat: Can eat in or take out, but take out predominates (e.g., Bakery, donut shop, ice cream shop, cookie shop, juice bar, bar/pub (for our purposes it’s a case by case, generally 7 if food can be ordered and served to you by waiter/bartender), pretzel shop, cinnamon roll shop, and bagel shops).

10. Mobile Food/Vending (type 21)
Ready to eat food on wheels. If you see it, record it. Includes S7.

11. S13

12. S6 should be type-number 16, include Kmart, Target, and Walmarts without full grocery stores within their stores

S14 and R30 should be coded case by case

S15 should be number 3, case by case if more towards convenience store than 1. Call/Google

R24 case by case 6 or 7

**1.** Glanz K, JF Sallies, BE Saelens, Frank L. Nutrition environment measures in stores (NEM-S) development and evaluation. *Am J Prev Med.* 2007;32(4):282-289.

**2.** Saelens BE, Glanz K, Sallis JF, LD F. Nutrition Environment Measures Study in restaurants (NEMS-R): development and evaluation. *Am J Prev Med.* 2007;32(4):273-281.
